# Emotional Stress Induced Broken Heart Syndrome: A Case Report

**DOI:** 10.7759/cureus.32384

**Published:** 2022-12-10

**Authors:** Lim Wei Juan, Neerusha Kaisbain

**Affiliations:** 1 Cardiology, National Heart Institute (IJN), Kuala Lumpur, MYS; 2 Cardiology, Hospital Queen Elizabeth II, Kota Kinabalu, MYS

**Keywords:** minoca, stress cardiomyopathy, broken heart syndrome, takotsubo syndrome, covid-19

## Abstract

Takotsubo syndrome (TTS) is a medical condition mostly due to emotional or physical stress which frequently leads to misdiagnosis or late diagnosis. Patients tend to present initially with acute heart failure or acute coronary syndrome to our emergency department. Here we describe a patient with no history of cardiovascular disease, who developed TTS due to emotional stress from the death of her husband and then fully recovered during follow-up.

## Introduction

Takotsubo syndrome (TTS), also known as stress cardiomyopathy or broken heart syndrome, is a morbid condition thought to be triggered by emotional or physical stress leading to cardiac endothelium or microvascular dysfunction [1]. Here we describe a case of TTS that was accurately diagnosed by cardiac magnetic resonance imaging (MRI).

## Case presentation

A 52 years old postmenopausal woman with no significant medical illness apart from being overweight (BMI 26.7kg/m^2^) presented to our emergency department for left-sided chest pain. It was dull-aching in nature and non-radiating. The pain lasted for ten minutes and it was relieved by rest. It was associated with difficulty in breathing but there was no profuse sweating. Blood pressure was 110/70 with a heart rate of 76 and the oxygen saturation at room air was 97%. Physical examination was unremarkable and no murmur was heard. Further history also noted patient husband passed away recently due to COVID-19 infection.

**Figure 1:**
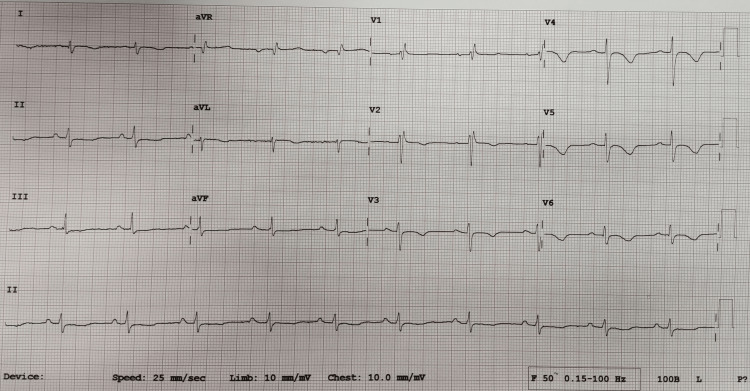
Electrocardiogram was done showed T wave inversion in lead I, aVL, V2 to V5

**Figure 2:**
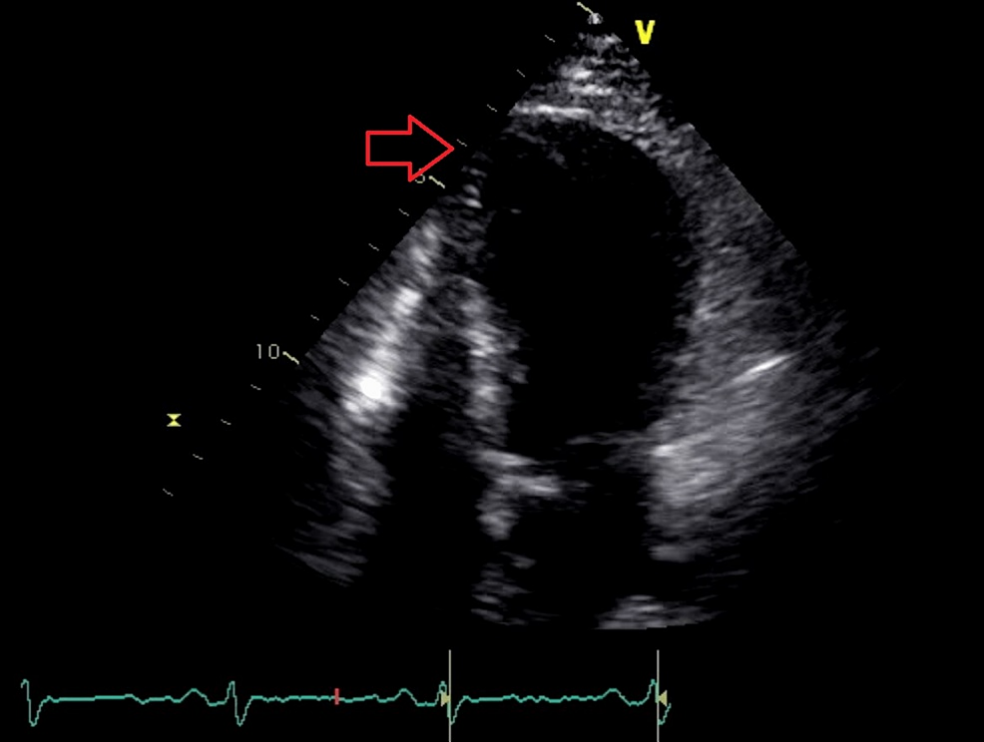
Transthoracic echocardiogram showed apical hypokinesia with ballooning (red arrow)

**Figure 3:**
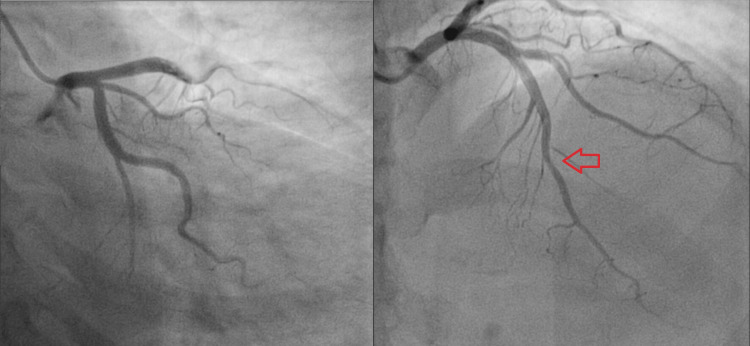
Normal left circumflex artery with mild disease at mid-segment left anterior descending artery (red arrow)

**Figure 4:**
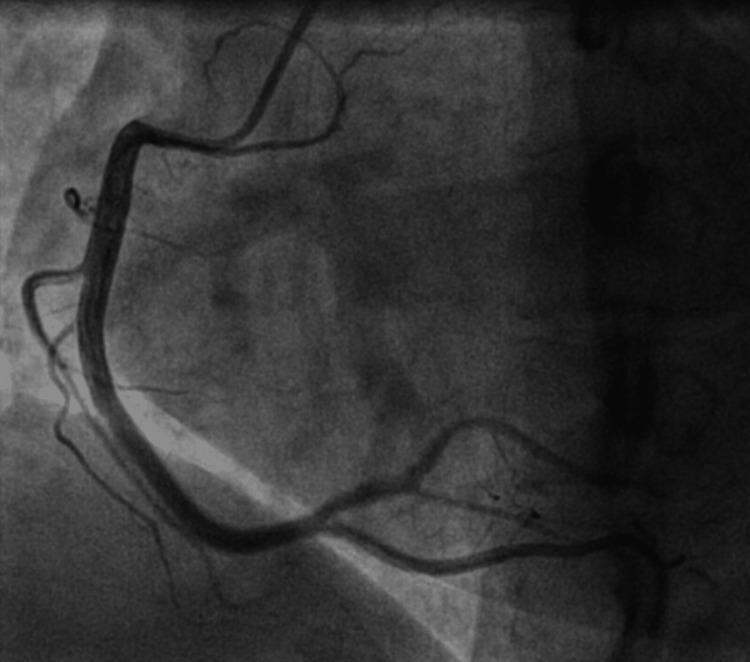
Normal right coronary artery

Blood investigation was significant for raised cardiac troponin T of 35ng/L; and N-terminal-prohormone B-type Natriuretic Peptide (NT-proBNP) of 1346pg/ml. Severe acute respiratory syndrome coronavirus 2 (SARS-CoV-2) reverse transcriptase polymerase chain reaction (PCR) nasopharyngeal swab test was negative which excluded COVID-19 infection. Electrocardiogram (ECG) showed sinus rhythm, normal axis, and T wave inversions in lead I, aVL, V2 to V5 (Figure 1). Transthoracic echocardiogram showed left ventricular dysfunction with an ejection fraction of 38% and severe mid to apical hypokinesia (Figure 2). Taking into account the history of chest pain with positive troponin T and regional wall motion abnormalities (RWMA), a diagnosis of non-ST segment elevation myocardial infarction (NSTEMI) was made. Coronary angiogram was performed which showed normal left main artery, left circumflex artery, and right coronary artery. There was only mild disease at mid-segment left anterior descending artery which did not explain the significantly reduced ejection fraction with RWMA (Figures 3, 4).

**Figure 5:**
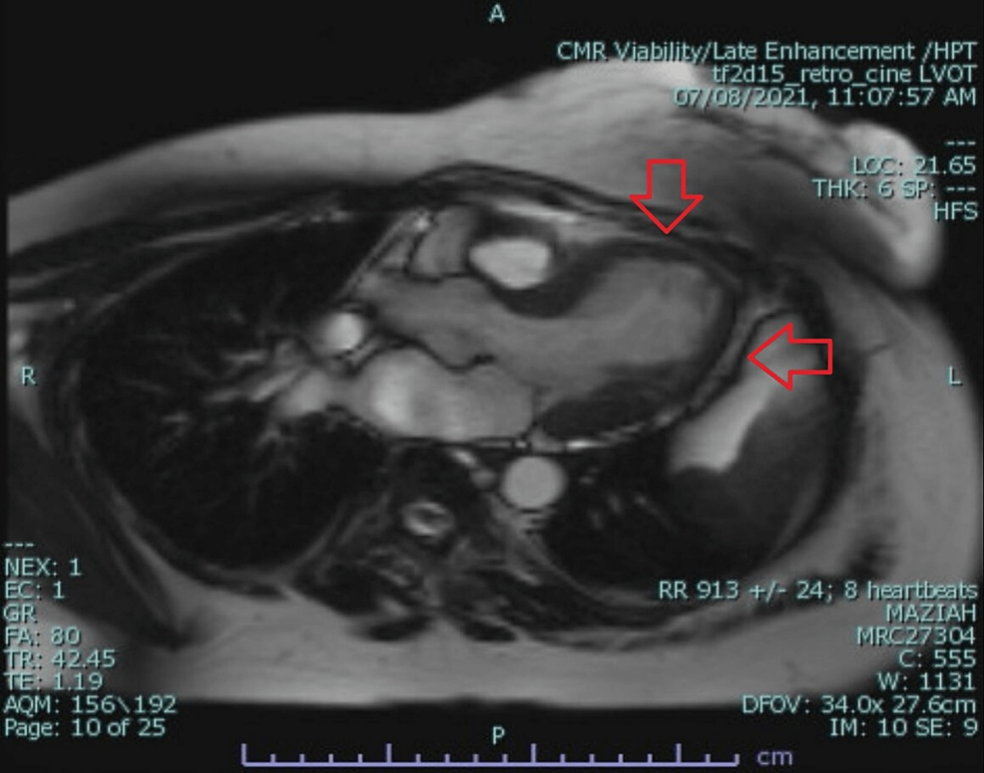
Cardiac magnetic resonance imaging (MRI) showed mid to apical severe hypokinesia with ballooning of the apical segment (red arrow)

Cardiac MRI was done to investigate other causes of myocardial infarction (MI) with non-obstructive coronary arteries (MINOCA). Cardiac MRI showed mid to apical severe hypokinesia with ballooning of the apical segment which is consistent with Takotsubo cardiomyopathy. There was no myocardial fibrosis, infarction, infiltration, or late gadolinium enhancement (LGE) (Figure 5).

During her stay in the hospital, she was started on antiplatelet, statin along with evidence-based heart failure medications such as sacubitril/valsartan (Entresto), bisoprolol, empagliflozin, and spironolactone. After performing a cardiac MRI and the patient was informed of the findings, she was discharged with medications and a clinic appointment to repeat the echocardiogram. The patient was also referred to a counselor for counseling.

## Discussion

In clinical practice, approximately 6 to 8% of patients presenting with ST elevation myocardial infarction (STEMI) and NSTEMI will have normal or nonobstructive coronaries [2]. In the past, with the limitation of investigation to ECG, cardiac enzymes, and coronary angiography in patients presenting with an acute coronary syndrome, clinicians were often left puzzled with the underlying cause of such presentation, and many resorted to the end diagnosis of NSTEMI or STEMI [2]. This resulted in patients being placed on long-term secondary prophylaxis treatments [2]. Some dismissed the presentation as false positive MI and the case is closed with no further investigations performed [3]. This may result in dire consequences, as we now know they are not benign after all [4]. They have a lower survival rate as compared to the normal population. Consequently, the term myocardial infarction with non-obstructive coronary arteries (MINOCA) was coined [3].

According to the European Society of Cardiology non-ST segment elevation acute coronary syndrome (ESC NSTE-ACS) 2020 guidelines, MINOCA is clinically defined as the presence of acute myocardial infarction (AMI) based on the “Fourth Universal Definition of Myocardial Infarction” criteria; absence of obstructive coronary artery disease (>50% stenosis in any major epicardial vessels) and no overt cause for the clinical presentation at the time of angiography, e.g. sepsis, pulmonary embolism, and myocarditis [4,5]. In MINOCA, the underlying mechanism is essentially ischemic in origin [1]. However, there are several non-ischemic conditions where there is myocardial injury and its presentation mimics that of AMI [2]. These include myocarditis, cardiomyopathy, and pulmonary embolism [2]. MINOCA is a broad umbrella term and is used as a working diagnosis just like heart failure, which requires the clinician to perform further investigations to determine the underlying cause [6]. The potential causes of MINOCA can broadly be divided into coronary causes and non-coronary causes with non-coronary causes including cardiac and extracardiac disorders [3,5]. Examples of coronary causes are coronary spasm, coronary microvascular dysfunction, plaque disruption, and coronary dissection; non-coronary cardiac causes such as TTS and other cardiomyopathies [5]. MINOCA tends to occur more in females and those of the younger age groups, and is also more prevalent in those with NSTEMI, just like the case we described [5].

Cardiac MRI should be the initial investigation to look for cardiac causes of MINOCA [7]. It can reveal the underlying diagnosis, e.g. TTS, or reclassify a presumed diagnosis of MI, e.g. myocarditis [2]. In 87% of the time, patients who were labeled as MINOCA and who underwent cardiac MRI in a timely manner were diagnosed with myocarditis instead [2]. Other cardiac causes of MINOCA which can be diagnosed via cardiac MRI include TTS, hypertrophic or dilated cardiomyopathy [2]. Apart from cardiac MRI, coronary vascular imaging is also used to determine the cause of MINOCA, namely, intravascular ultrasound (IVUS) and optical coherence tomography (OCT), which can aid in diagnosing coronary causes of MINOCA, namely, plaque disruption or coronary dissection [8].

Our patient underwent coronary angiography and it revealed non-obstructive coronaries. As per the guideline recommendation, a cardiac MRI was performed which revealed TTS. TTS was first described by Sato et al. in 1990 in Japanese textbook [9]. Takotsubo means octopus trap in Japanese, and this name was derived due to the shape that the left ventricle assumes at the end of systole [10]. Also described as “broken heart syndrome”, “stress cardiomyopathy”, and “apical ballooning syndrome”, TTS’s hallmark is its association with preceding emotional and physical triggers, although it can still occur without one [11].

As per InterTAK (international takotsubo) Diagnostic Criteria, our patient who is postmenopausal has left ventricular dysfunction with mid to apical focal wall motion abnormalities, ECG abnormalities (T-wave inversion in V2-V5), elevated cardiac markers (hs troponin T, NT-pro-BNP) without significant coronary artery disease and myocarditis, preceded by emotional trigger [10]. Current literature shows that postmenopausal females are at higher risk to develop TTS due to the loss of the sympatholytic effect of estrogen with increased vascular and myocardial response to beta-adrenergic receptors [12].

The treatment of TTS is mainly based on expert consensus, owing to the lack of randomized controlled trials in this population [13]. The use of angiotensin-converting enzyme inhibitors (ACEi) or angiotensin II receptor blockers (ARB) showed improved survival [13]. In general, TTS is a reversible syndrome, but life-threatening complications may occur during the acute phase of hospitalization, such as ventricular arrhythmias, acute ventricular septal defect, necessitating close monitoring [13]. This case report is to raise awareness that the absence of obstructive coronary disease should not give false reassurance to clinicians that MI has not occurred [14]. Instead, it should trigger the clinician to perform further investigations to arrive at a diagnosis.

## Conclusions

TTS is reversible and hence is important not to misdiagnose. Further investigations should be performed after the initial coronary angiogram showed non-obstructive coronary arteries in patient with NSTEMI. Cardiac MRI is one of the important imaging modalities for the workup of MINOCA. TTS has a good prognosis with favorable recovery.
